# Working online or offline during COVID-19: Which has more impacts on stock price?

**DOI:** 10.3389/fpsyg.2022.970961

**Published:** 2022-12-15

**Authors:** Wanjiao Jia, Yuanyuan Xu, Xiaowu Lu

**Affiliations:** ^1^School of Management, Shanghai University, Shanghai, China; ^2^School of Accounting, Shanghai Lixin University of Accounting and Finance, Shanghai, China

**Keywords:** online work, offline work, analyst visits, information environment, stock prices

## Abstract

Remote work has become increasingly popular and important after the spread of COVID-19, but its impact on the financial market is in dispute. Using a unique dataset of analyst visits in China and multiple regression, we examine the impact of remote work on the financial market by comparing the market reaction to analysts’ online and offline visits. Results show that online visits have a significantly greater impact on stock prices than offline visits, as discussion depth, information sharing, and information dissemination are enhanced. Additionally, online visits can predict the changes in funds’ holdings and firms’ future performance. Overall, our findings suggest that remote work improves the information environment of the financial market during COVID-19.

## Introduction

With the development of technology and the outbreak of COVID-19, remote work has become an increasingly important and prevalent way of working (Brynjolfsson et al., 2020). For instance, 69% of US employees worked remotely at the peak of the pandemic, while only 4.1% of them telecommuted half-time or more before the pandemic (Global Workplace Analytics, 2022). The case is quite similar in Canada, the percentage of employees working from home is 32% at the beginning of 2021 and only 4% in 2016 (Mehdi and Morissette, 2021). Most previous literature focuses on the impact of remote work on employees’ and managers’ behaviors (Allen et al., 2015; Bloom et al., 2015; Felstead and Henseke, 2017; Parker et al., 2020), but research about its impact on the financial market is limited. It is not clear whether and how remote work during COVID-19 impacts the information environment of the financial market.

**Figure 1:**
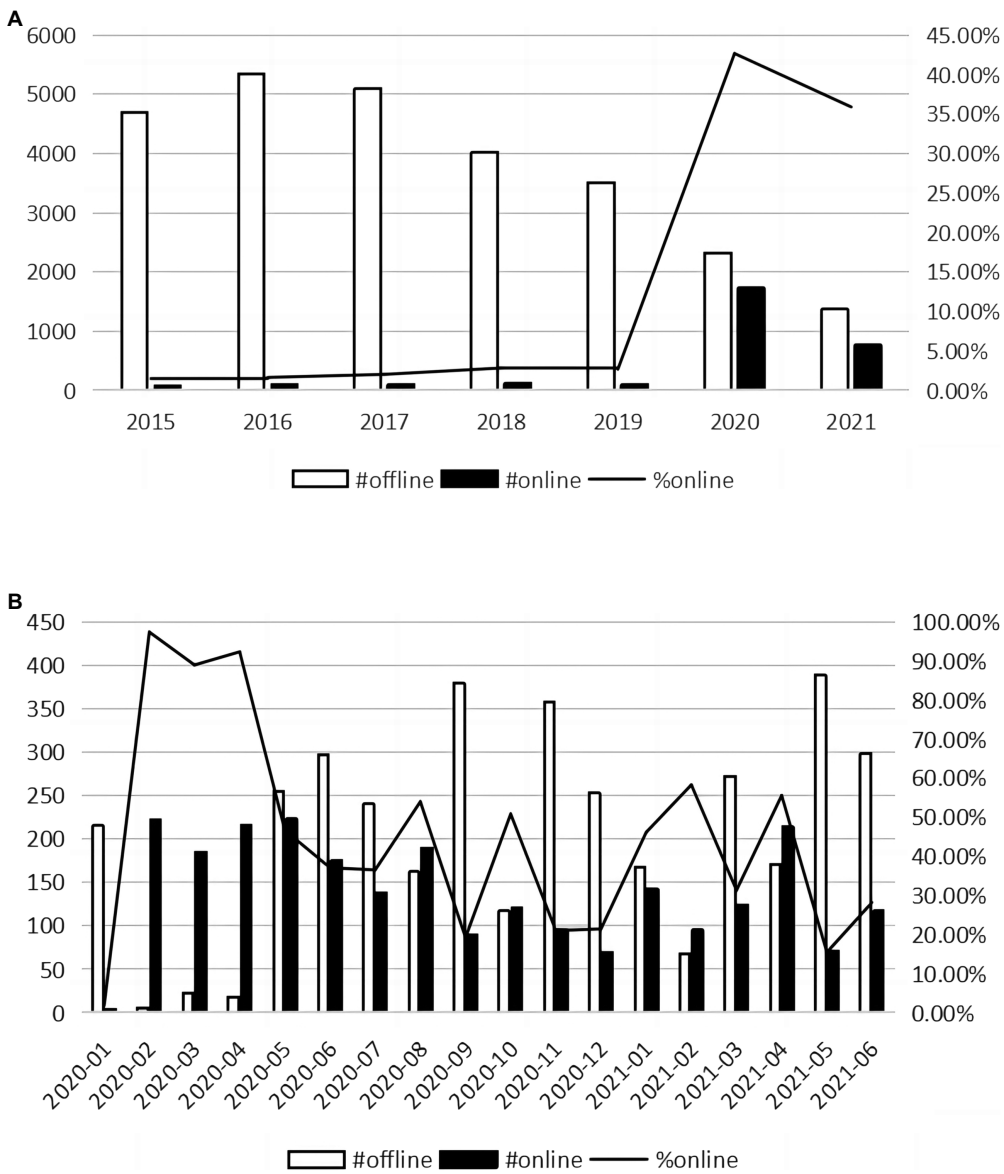
The distribution of analysts’ online and offline visits: **(A)** Distribution by year **(B)** Distribution by month.

As an important information intermediary in the financial market, financial analysts’ information acquisition activities affect stock prices (Green et al., 2014; Cheng et al., 2019). Analysts’ private access to management is an important information acquisition way for financial analysts (Brown et al., 2015; Bushee et al., 2018; Han et al., 2018). Before the pandemic, analysts usually access management through offline visits, which refer to analysts’ field trips to corporate headquarters and production facilities. But the COVID-19 pandemic disrupts analysts’ offline site visits. Many governments adopted strict social quarantine policies to prevent the spread of COVID-19, especially during the beginning period. With field trips restricted, analysts actively adopted online visits, which refer to analysts’ non-face-to-face communication with management through telephone, Internet, etc., a kind of remote work, to collect information during the COVID-19 crisis. Taking China as an example, the percentage of analysts’ online visits increased from 3.09% in 2019 to 42.66% in 2020 and the percentage is especially high during the first 3 months (Figure 1). As China is one of the countries with the strictest epidemic control policies, COVID-19 has brought the greatest exogenous impact on analysts’ offline visits in China. Taking advantage of analysts’ visit data disclosed by firms listed in the Shenzhen Stock Exchange (SZSE) in China, we investigate the impact of analysts’ remote work on the financial market by comparing the impact of analysts’ online and offline visits on stock prices and explore the mechanisms that affect the effect of analysts’ remote work.

From the perspective of information acquisition, offline visits have more advantages in getting first-hand information through field visits and face-to-face communication (Short et al., 1976; Cheng et al., 2016). But the communication condition is better during online visits for the following three reasons. First, online visits are less likely to be affected by reception activities and are more task-oriented, which makes communication efficiency and discussion depth improved. Second, social class pressure is lower during online visits, which encourages disadvantaged visitors to join the communication instead of just listening and enhances information sharing (Kiesler et al., 1984; Dubrovsky et al., 1991). Third, the low participating cost of online visits attracts more investors to participate and increases the information diffusion effect (Hwang et al., 2019). Hence, online or offline visits, which have more impact on stock price is an empirical question. It is not only theoretically interesting but also practically important, especially after the outbreak of COVID-19.

Our results indicate that analysts’ remote work improves the information environment of the financial market and decreases the damage of COVID-19. We find that analysts’ online visits have a significantly greater impact on stock prices than offline visits during COVID-19. The results are robust to several robustness checks including changing event windows, controlling for visit characteristics, and considering other big events’ impacts and the treatment effect. We also find that questions and answers are longer during online visits, online visits attract more institutional investors to participate, and the advantage of online visits is more pronounced when the number of visitors is higher. These are three possible mechanisms through which analysts’ online visit, a kind of remote work, plays its role. Furthermore, we find that online visits can predict the changes in funds’ holdings and firms’ future performance.

Our study contributes to the literature in four important ways. First, this paper enriches the literature on the impact of remote work on financial markets. Cai et al. (2022) and Gao et al. (2020) find that online board meetings and online annual meetings can improve corporate governance, indicating remote work positively impacts the financial market. But Li et al. (2021b) find analysts’ forecasts are less accurate after online visits than after offline visits, suggesting remote work is inferior. However, their conclusion could be biased for two reasons. For one thing, analysts’ forecasts only reflect analysts’ beliefs, which may be different from the overall market participants’ beliefs. For another, only a small portion of analysts issue forecasts after corporate visits, so forecast accuracy may not be a good measure to capture information content of online and offline visits. In our sample, only 12.6% of analysts issued earning forecasts within 30 days after corporate visits. Thus, to understand the impact of online work, it is important to investigate whether there are differences in stock price reactions between online and offline visits, as stock price reflects the changes in all market participants’ beliefs. From the perspective of market reaction, our results support that analysts’ remote work has a positive impact on the financial market and extends the literature about the economic consequences of remote work.

Second, this paper contributes to studies of COVID-19’s impact on financial markets. Prior literature mainly focuses on the negative impact of COVID-19 on stock markets (Liu et al., 2020; Contessi and De Pace, 2021; Mazur et al., 2021), and tries to find safe havens against stock markets during the pandemic (Salisu et al., 2021; Yousaf et al., 2021; Yousaf and Ali, 2021; Mokni et al., 2022). However, few studies offer direct empirical evidence on what channels can reduce the negative impact of COVID-19 on financial markets. We provide new insights into the relationship between remote work and the information environment by studying an important information acquisition channel for analysts during the COVID-19 crisis, that is, online visits.

Third, our study has implications for understanding the mechanisms through which analysts’ remote work affects the information environment of the financial market. Our analyses imply that online visits deepen discussion between analysts and managers, facilitate information sharing among visitors, and amplify information dissemination.

Finally, to our knowledge, this paper is among the early studies that focus on analysts’ online private information acquisition. The literature on analysts’ information acquisition activities mainly focuses on conference calls (Mayew et al., 2013), private interactions with managers (Soltes, 2014), broker-hosted investor conferences (Green et al., 2014), and offline visits (Cheng et al., 2016). Our findings show that online private visits provide analysts with a more efficient way to acquire information than offline visits during COVID-19.

The remainder of this study proceeds as follows: Section 2 introduces the related literature and develops a hypothesis; Section 3 explains data sources, main variables, and the research model; Section 4 provides empirical results; Section 5 discusses further analyses; and Section 6 concludes.

## Literature review and hypothesis development

### Literature review

#### The COVID-19 impacts on financial markets

COVID-19 has a significant impact on financial markets. The emerging literature mainly focuses on two aspects, that is, the negative impact of COVID-19 on stock markets and the safe havens that can hedge risks of stock markets during the COVID-19 crisis. COVID-19 is the key factor causing volatility in the stock market in the early beginning of 2020 (Liu et al., 2020; Li et al., 2021a; Okorie and Lin, 2021), especially in emerging markets and for small firms (Al-Awadhi et al., 2020; Topcu and Gulal, 2020; Harjoto et al., 2021). And the negative impact on diverse industries’ stock markets is different (Mazur et al., 2021). But the negative impact of COVID-19 on emerging stock markets has gradually fallen and begun to taper off by mid-April, 2020 (Topcu and Gulal, 2020). With regard to the literature exploring safe havens during the COVID-19 crisis, previous papers mainly explore the return and volatility transmission in different financial markets, such as the cryptocurrency markets (Yousaf and Ali, 2020), gold markets (Salisu et al., 2021), bond markets (Ali et al., 2022), and metal and energy markets (Yousaf, 2021). These studies show that COVID-19 influences information transmission in different markets. However, prior studies ignore the potential effect of remote work by information intermediaries, such as financial analysts, on the dissemination of information during COVID-19.

#### Remote work

Remote work greatly impacts people’s life and society. But most articles studying the consequences of remote work focus on employees’ and managers’ behaviors (Allen et al., 2015; Bloom et al., 2015; Felstead and Henseke, 2017; Parker et al., 2020). Remote work leads to a variety of positive outcomes such as high organizational commitment, employee productivity, job satisfaction, and low turnover of staff (Baltes et al., 1999; Golden, 2006; Bloom et al., 2015), but these benefits come at the cost of less promotion rate conditional on performance, work intensification and a greater inability to balance work and life (Bloom et al., 2015; Felstead and Henseke, 2017). Remote work also has negative effects on social and professional isolation, relationships with coworkers and supervisors, knowledge sharing, and innovation (Feldman and Gainey, 1997). A substantial number of managers distrust workers and lack the capability to lead remotely (Parker et al., 2020).

Recently, some scholars began to focus on the effect of remote work on financial markets. For example, Gao et al. (2020) find that online annual shareholder meetings significantly increase the participation of shareholders, especially minority shareholders. Cai et al. (2022) find that by facilitating status equalization among attendees and alleviating their pressure for conformity, remote meetings improve board monitoring effectiveness.

As an important information intermediary, financial analysts generally do not conduct remote work to collect private information before COVID-19, because offline visit is an effective way to obtain first-hand information. The outbreak of COVID-19 forced analysts to collect private information through online visits. Therefore, analysts’ information acquisition activity during COVID-19 is a good setting to study the effectiveness of remote work in financial market.

### Hypothesis development

Visits are an important type of private information acquisition activity for analysts in the financial market (Cheng et al., 2019). However, after the outbreak of COVID-19, online visits replaced offline visits and became the main channel for analysts to collect information.

Both online and offline visits have their advantages and limitations. On the one hand, online visits have limitations in getting first-hand information compared to offline visits. Firstly, seeing is believing. Compared to offline visits, analysts cannot field visit corporate headquarters and production facilities, talk to employees, and have in-person communication with managers during online visits, which is helpless for analysts to clearly understand the business models and corporate culture of the company (Cheng et al., 2016). Moreover, online visits cannot obtain non-verbal information through management’s facial expressions and body language, which are important information clues. Short et al. (1976) found that online communication is inferior to face-to-face communication on the side of information richness and social presence due to the lack of information clues. Daft and Lengel (1984) also found that communication media can affect participants’ response speed to information and personalized expression. Finally, as China is a relational society, face-to-face interaction is a good way to develop relationships (Barry and Fulmer, 2004), which is useful for analysts to obtain private information. However, online visits have no such advantage.

But on the other hand, online visits have more effective communication conditions. First, online visits are more likely to be task-oriented, which can improve communication efficiency and discussion depth. Condon and Cech (1996) found that task orientation in communication is an important determinant of communication efficiency. During offline visits, company management needs to spend energy arranging analysts’ travel, catering, and other activities, which reduces task orientation in communication. But during online visits, companies do not need to spend much time and energy on such affairs. With less interference from other affairs, management is more likely to focus on the communication contents.

Second, online visits can help the disadvantaged ones to communicate more efficiently and enhance information sharing. In-person communication puts social class pressure on disadvantaged ones, such as young and inexperienced analysts. Online visits can reduce participants’ social class pressure, and enable the disadvantaged to express their doubts and opinions more effectively (Kiesler et al., 1984; Dubrovsky et al., 1991). In this regard, online visits increase the communication efficiency of all participants.

Finally, online visits have a stronger information diffusion effect. Compared with the high cost of offline visits, the low participating cost of online visits can attract more investors to participate, which amplifies information dissemination to the market (Hwang et al., 2019).

Therefore, online and offline visit which has more impact on stock price is an empirical question. We propose the hypothesis in null form.

*H1*: There is no difference in stock price movements between analysts’ online visits and offline visits.

## Research design

### Sample and data

Our sample consists of all visits conducted by financial analysts to listed companies in SZSE from January 2020 to June 2021. We do not include analysts’ visits to listed companies in Shanghai Stock Exchange (SHSE), because SHSE only requires listed companies to report to SHSE and these data are not publicly available. We choose January 2020 as the start month for two reasons. First, COVID-19 started in Wuhan City in December 2019 and spread out in January 2020, which means COVID-19 started to impact the whole country from January 2020. Second, the proportion of analysts’ online visits increased sharply in February 2020 (as shown in Figure 1), so we believe that including samples before 2020 may bias the results. According to the Fair Disclosure rule of SZSE, listed companies should disclose visiting information within two workdays after the visit. So we delete visits whose disclosure date is before the visit date and visits disclosed four natural days after the visit date to ensure the accuracy of visit date information. Then, we drop visits without data for the estimation of abnormal stock returns. Finally, we eliminate visits with missing control variables in the multivariate regressions of market reaction. Our final sample consists of 4,020 visits to 937 firms in 1,308 firm-years. All data are from China Stock Market Accounting Research (CSMAR) database^1^ except that fund ownership data are from the Wind database. Table 1 reports our sample selection criteria.

**Table 1:** Sample selection criteria.

	# of visit events	# of firms	# of firm-years
(1) All private meetings of listed companies in SZSE with analyst participation from January 2020 to June 2021	6,180	1,146	1,693
(2) After deleting private meetings without data for the estimation of abnormal stock returns	4,406	966	1,369
(3) After deleting private meetings with missing control variables in the multivariate regressions of market reaction	4,020	937	1,308

### Model and variables

To test the different stock price impacts of online and offline visits, we construct Model 1 with firm and year fixed effects included.


(1) $$\begin{array}{l} \mathrm{ABN}\_\mathrm{ABSAR}\left(0,1\right)=\alpha_{1}\times \mathrm{ONLINE}+\beta \times \mathrm{Controls}+\gamma \\ \times \;\mathrm{Fixed Effects}+\varepsilon \end{array}$$

In Model 1, following previous studies (Bushee et al., 2011; Cheng et al., 2019), we measure the stock price impact of visits using the standardized absolute value of abnormal returns (*ABN_ABSAR(0,1)*) in the 2-day window around analyst visits, that is, the (0, +1) window, where portfolio return is calculated with equal weight. The detailed calculation is in the Appendix. *ONLINE* is a dummy variable that equals one when the analyst visit is conducted online, and zero otherwise. To ensure the measure of *ONLINE* is pure, any online visit combined with offline visit is assigned to the offline visit group, i.e., *ONLINE* is zero in this case. We note that such measurement may bias the results against the prediction that analysts’ online visits have more impact on stock prices.

We also control for three sets of variables that might affect the stock price impact of analysts’ visits. First, we include abnormal return from 1 year prior to the analyst visit until 30 days before the visit (*ABRET*), absolute abnormal return from 1 month prior to the visit until 11 days before the online visit (*ABRET_pre_visit*), and average monthly share turnover for the year prior to the visit until 30 days before the visit (*TURNOVER*) to alleviate the concern that the market reaction is confounded by the information events occurring right before analyst visits. Second, we include the market-to-book ratio (*MB*), the change in net income (*∆EPS*), and sales growth (*SGROWTH*) to capture firm profitability. Thirdly, market value (*LOGMV*), firm leverage (*LEVERAGE*), stock beta (*BETA*), and firm age (*LOGAGE*) are included to capture firm risk. The Appendix presents variable definitions.

## Empirical analyses

### Descriptive statistics

Table 2 presents the descriptive statistics of the main variables. The mean of *ABN_ABSAR(0,1)* is 0.280, indicating that analyst visits lead to strong market reactions. The mean of *ONLINE* is 0.405, suggesting that 40.5% of analyst visits are conducted online in our sample period. The visited firms on average have positive stock return (*ABRET*) of 21.6%, monthly share turnover rate (*TURNOVER*) of 11.8%, market-to-book ratio (*MB*) of 3.381, positive change in net income (24.8% of prior year net income), annual sales growth of 17.8% of total assets, leverage of 41.5%, and beta of 0.997. The visited firms on average have been listed for 9 years.

**Table 2:** Descriptive statistics.

Variables	*N*	mean	SD	min	p25	p50	p75	max
ABN_ABSAR(0,1)	4,020	0.280	1.477	−1.273	−0.682	−0.163	0.685	6.819
ONLINE	4,020	0.405	0.491	0.000	0.000	0.000	1.000	1.000
ABRET	4,020	0.216	0.736	−0.855	−0.257	0.053	0.479	3.631
ABRET_pre_visit	4,020	0.117	0.107	0.002	0.042	0.088	0.156	0.565
TURNOVER	4,020	0.118	0.076	0.022	0.063	0.100	0.151	0.400
MB	4,020	3.381	2.567	0.620	1.794	2.700	4.065	15.625
LOGMV	4,020	16.061	0.931	14.505	15.364	15.933	16.604	18.881
LOGCOVERAGE	4,020	2.440	1.471	0.000	1.386	2.708	3.638	4.898
LEVERAGE	4,020	0.415	0.175	0.080	0.282	0.414	0.529	0.920
∆EPS	4,020	0.248	2.312	−10.205	−0.216	0.150	0.530	11.811
SGROWTH	4,020	0.178	0.383	−0.425	0.017	0.128	0.252	2.923
BETA	4,020	0.997	0.212	0.476	0.848	0.992	1.144	1.531
LOGAGE	4,020	2.276	0.559	1.099	1.792	2.398	2.639	3.332

This table presents descriptive statistics on the variables used in our main analysis. All variables except for the dummies are winsorized at the 1st and the 99th percentiles.

### Multivariate analyses

Column (1) of Table 3 reports the main regression result. The coefficient on *ONLINE* is not only statistically significant at 1% level, but also economically significant. Given the average value of *ABN_ABSAR(0,1)* is 0.280 in our sample, the price impact of online visits is 0.300 higher than that of offline visits, about 3 times (0.4585/0.1585) offline visits’ market reaction. *ABN_ABSAR(0,1)* in column (2) is calculated with value-weighted portfolio return and the result is robust. Our results suggest that analysts’ online visits have greater market impacts and support that online visits are more informative than offline visits during COVID-19. That implies that online visit is a more useful information acquisition channel for analysts during COVID-19, and analysts’ online visits can make the stock price more effectively reflect the value of companies.

**Table 3:** The difference between market reaction of online and offline visits.

	(1)	(2)
	ABN_ABSAR(0,1) equal-weighted	ABN_ABSAR(0,1) value-weighted
ONLINE	0.300***	0.296***
	**(3.91)**	**(3.94)**
ABRET	−0.167	−0.146
	(−1.49)	(−1.45)
ABRET_pre_visit	−0.324	−0.317
	(−0.92)	(−0.93)
TURNOVER	7.571***	7.352***
	(4.73)	(4.78)
MB	−0.046	−0.047
	(−0.69)	(−0.74)
LOGMV	−0.148	−0.099
	(−0.47)	(−0.33)
LOGCOVERAGE	0.068	0.071
	(0.79)	(0.85)
LEVERAGE	−0.669	−0.867
	(−0.56)	(−0.73)
∆EPS	−0.038	−0.038
	(−1.56)	(−1.56)
SGROWTH	0.008	0.032
	(0.05)	(0.20)
BETA	0.851**	0.825**
	(2.24)	(2.18)
LOGAGE	0.788	0.447
	(0.57)	(0.33)
_cons	−0.659	−0.577
	(−0.11)	(−0.10)
FIRM	YES	YES
YEAR	YES	YES
*N*	4,020	4,020
*R* ^2^	0.313	0.314

Standard errors are adjusted for firm-level clustering. *T*-statistics are presented in the parentheses. ***, **, * indicate statistical significance at the 1, 5, and 10% levels, respectively. The bold represents in ABN_ABSAR(0,1) equal-weighted in column (1) is calculated with equal-weighted portfolio return and ABN_ABSAR(0,1) equal-weighted in column (2) is calculated with value-weighted portfolio return.

The results for the control variables suggest that the stock price impact of analyst visits is larger for firms with higher prior turnover (*TURNOVER*) and higher stock beta (*BETA*). Other control variables are not significant, which is different from Cheng et al. (2019). That may be because our sample only lasted one and a half years, and we control firm fixed effect instead of industry fixed effect.

### Robustness checks

In this section, we do three robustness checks to alleviate the concern that the results reported in Table 3 are driven by investors’ herding behavior and other important events that happened around analyst visits. We employ three methods to address this concern, including using longer event windows, adding visit characteristics as control variables, and controlling other big events. We also use a two-step model to consider possible treatment effect.

#### Using longer event windows

If the greater impact of online visits on stock prices is due to investors’ herding behavior but not the information content of the visits^2^, we would observe the difference decline soon after. In that case, we may not observe a significant impact of *ONLINE* on *ABN_ABSAR* when it is measured in the 3-day, 4-day, and 5-day window around analyst visits, that is, the [0, +2], [0, +3], and [0, +4] window. In Table 4, we measure *ABN_ABSAR* in the 3-day, 4-day, and 5-day window around analyst visits, with the portfolio market return calculated with equal weight. All three coefficients of *ONLINE* are significantly positive, indicating that the different market impacts of online and offline visits still exist 4 days after the visits, which mitigates the concern of investors’ herding behavior.

**Table 4:** Robustness check - change windows.

	ABN_ABSAR(0,2)	ABN_ABSAR(0,3)	ABN_ABSAR(0,4)
	(1)	(2)	(3)
ONLINE	0.285***	0.228**	0.151*
	(3.14)	(2.46)	(1.66)
ABRET	−0.173	−0.162	−0.154
	(−1.64)	(−1.36)	(−1.38)
ABRET_pre_visit	−0.725	−0.873	−0.987
	(−1.51)	(−1.47)	(−1.34)
TURNOVER	9.876***	8.538***	8.970***
	(4.47)	(4.62)	(4.70)
MB_t-1_	−0.009	−0.015	−0.025
	(−0.16)	(−0.25)	(−0.43)
LOGMV_t-1_	−0.420	−0.435	−0.257
	(−1.19)	(−1.28)	(−0.65)
LOGCOVERAGE	0.065	0.032	0.038
	(0.61)	(0.30)	(0.33)
LEVERAGE_t-1_	−1.048	−0.935	0.747
	(−0.88)	(−0.70)	(0.57)
∆EPS_t-1_	−0.035	−0.016	0.036
	(−0.96)	(−0.42)	(0.86)
SGROWTH_t-1_	0.129	0.043	0.108
	(0.70)	(0.22)	(0.61)
BETA	1.073**	1.300***	0.872*
	(2.38)	(2.84)	(1.74)
LOGAGE	1.076	1.758	0.904
	(0.73)	(1.13)	(0.58)
_cons	2.685	1.409	0.194
	(0.42)	(0.21)	(0.03)
FIRM	YES	YES	YES
YEAR	YES	YES	YES
*N*	4,020	4,020	4,020
*R* ^2^	0.320	0.346	0.328

Control variables are as the same in Table 2. Standard errors are adjusted for firm-level clustering. *T*-statistics are presented in the parentheses. ***, **, * indicate statistical significance at the 1, 5, and 10% levels, respectively. ABN_ABSAR(0,2), ABN_ABSAR(0,3), and ABN_ABSAR(0,4) are calculated using the window (0,+2), (0,+3), and (0,+4).

#### Controlling visit characteristics

We also control for additional variables as Cheng et al. (2019) find that visit characteristics can affect market reactions. Table 5 presents the robust analyses controlling for the number of questions asked during a visit (*QUESTION_num*), the number of institutional investors participating in a visit (*INSTITUTION_num*), and three dummy variables (*CEO*, *CFO*, and *BOARDSEC*) that identify whether the CEO, CFO or board secretary attends a visit. The coefficients of *ONLINE* do not become smaller in magnitude or less significant, compared to the result in column (1) of Table 3. That means our conclusion is not affected by visit characteristics but lies in other advantages of online visits compared to offline visits. *INSTITUTION_num* and *BOARDSEC* are positive at 10% level, indicating that analyst visits with more institutional visitors and board secretary participation contain more information contents.

**Table 5:** Robustness check - control visit characteristics.

	ABN_ABSAR(0,1)
	(1)				(2)	(3)	(4)
ONLINE	0.299***	0.253***	0.291***	0.246***
	(3.90)	(3.10)	(3.76)	(2.99)
ABRET	−0.167	−0.171	−0.163	−0.167
	(−1.49)	(−1.52)	(−1.46)	(−1.49)
ABRET_pre_visit	−0.324	−0.338	−0.325	−0.337
	(−0.92)	(−0.96)	(−0.92)	(−0.96)
TURNOVER	7.543***	7.473***	7.612***	7.507***
	(4.70)	(4.65)	(4.76)	(4.67)
MB_t-1_	−0.046	−0.051	−0.047	−0.051
	(−0.68)	(−0.75)	(−0.70)	(−0.74)
LOGMV_t-1_	−0.149	−0.150	−0.140	−0.148
	(−0.47)	(−0.47)	(−0.45)	(−0.47)
LOGCOVERAGE	0.066	0.065	0.069	0.065
	(0.77)	(0.76)	(0.81)	(0.77)
LEVERAGE_t-1_	−0.658	−0.611	−0.725	−0.688
	(−0.55)	(−0.51)	(−0.61)	(−0.58)
∆EPS_t-1_	−0.037	−0.035	−0.036	−0.033
	(−1.55)	(−1.45)	(−1.49)	(−1.38)
SGROWTH_t-1_	0.007	0.004	0.011	0.005
	(0.05)	(0.02)	(0.07)	(0.03)
BETA	0.857**	0.872**	0.834**	0.859**
	(2.25)	(2.29)	(2.20)	(2.26)
LOGAGE	0.803	0.742	0.817	0.754
	(0.58)	(0.54)	(0.59)	(0.55)
QUESTION_num	0.006			0.001
	(0.51)			(0.11)
INSTITUTION_num		0.002*		0.002*
		(1.87)		(1.76)
CEO			−0.010	−0.033
			(−0.09)	(−0.30)
CFO			0.040	0.022
			(0.27)	(0.15)
BOARDSEC			0.199*	0.191*
			(1.76)	(1.67)
_cons	−0.720	−0.547	−0.957	−0.712
	(−0.12)	(−0.09)	(−0.16)	(−0.12)
FIRM	YES	YES	YES	YES
YEAR	YES	YES	YES	YES
*N*	4020.000	4020.000	4020.000	4020.000
*R* ^2^	0.313	0.315	0.314	0.316

Control variables are as the same in Table 2. Standard errors are adjusted for firm-level clustering. *T*-statistics are presented in the parentheses. ***, **, * indicate statistical significance at the 1, 5, and 10% levels, respectively. QUESTION_num is the number of questions asked. INSTITUTION_num is the number of all institutional visitors. CEO is an indicator variable that equals one if the CEO participates in a private meeting, and zero otherwise. CFO is an indicator variable that equals one if the CFO participates in a private meeting, and zero otherwise. BOARDSEC is an indicator variable that equals one if the board secretary participates in a private meeting, and zero otherwise.

#### Considering the timing of site visits

Given a firm has a visit, its timing can be endogenous. Analysts may visit a firm because it has recently had, or will soon have an important announcement. Following Cheng et al. (2019), we exclude visits around corporate earnings announcements and rerun Model 1. Based on that sample, following Hirshleifer et al. (2008) we further identify three variables that may potentially affect the timing of analyst visits and add them to the main regression. If the coefficients on ONLINE are still significant, then the timing of analyst visits is unlikely to have a significant impact on our inferences.

The sample size (3,334) in Table 6 is smaller than that in Table 3 because we exclude visits around earnings announcements. We add an indicator (*BIGEVENT*) for analyst visits that occur in the event window of major corporate events including mergers and acquisitions, seasoned equity offerings, right offerings, related party transactions, lawsuits, regulatory violations, and dividends in Column (2), an indicator for adjacent online visits (*ADJACENT*) in Column (3), the absolute abnormal returns on day −1 [*ABSAS(−1)*] in Column (4), and all three variables in Column (5). All coefficients of *ONLINE* in Table 6 are statistically significant, suggesting that our main inference still holds. In sum, these tests suggest that our main inferences are unlikely to be driven by the timing of analyst visits, at least based on the selected timing variables.

**Table 6:** Robustness check - Deleting private meetings around EA and control other factors.

		ABN_ABSAR(0,1)	
	(1)					(2)	(3)	(4)	(5)
ONLINE	0.314***	0.314***	0.300***	0.134*	0.127*
	(3.29)	(3.30)	(3.24)	(1.88)	(1.80)
ABRET	−0.139	−0.142	−0.134	−0.163*	−0.164*
	(−1.11)	(−1.13)	(−1.07)	(−1.95)	(−1.95)
ABRET_pre_visit	−0.393	−0.394	−0.410	−0.616	−0.627
	(−1.04)	(−1.02)	(−1.10)	(−1.51)	(−1.50)
TURNOVER	8.143***	8.221***	8.093***	5.062***	5.122***
	(4.92)	(4.93)	(4.91)	(4.08)	(4.13)
MB_t-1_	0.001	0.003	−0.004	0.036	0.036
	(0.02)	(0.07)	(−0.08)	(0.94)	(0.94)
LOGMV_t-1_	−0.241	−0.243	−0.236	−0.339	−0.338
	(−0.70)	(−0.71)	(−0.69)	(−1.27)	(−1.28)
LOGCOVERAGE	0.103	0.102	0.116	0.112	0.118
	(1.14)	(1.13)	(1.27)	(1.43)	(1.51)
LEVERAGE_t-1_	−0.371	−0.387	−0.333	0.147	0.150
	(−0.35)	(−0.36)	(−0.31)	(0.16)	(0.16)
∆EPS_t-1_	−0.033	−0.033	−0.034	−0.034*	−0.034*
	(−1.26)	(−1.23)	(−1.32)	(−1.65)	(−1.68)
SGROWTH_t-1_	0.021	0.019	0.025	0.020	0.019
	(0.13)	(0.11)	(0.15)	(0.13)	(0.13)
BETA	0.655	0.644	0.645	0.483	0.467
	(1.50)	(1.48)	(1.47)	(1.44)	(1.40)
LOGAGE	−0.392	−0.377	−0.433	0.332	0.324
	(−0.32)	(−0.31)	(−0.36)	(0.39)	(0.38)
BIGEVENT		−0.126			−0.132
		(−0.98)			(−1.32)
ADJACENT			0.152		0.082
			(1.46)		(1.13)
ABSAR(−1)				37.091***	37.040***
				(15.03)	(15.18)
_cons	3.184	3.196	3.141	2.623	2.612
	(0.54)	(0.54)	(0.53)	(0.58)	(0.58)
FIRM	YES	YES	YES	YES	YES
YEAR	YES	YES	YES	YES	YES
*N*	3,334	3,334	3,334	3,334	3,334
*R* ^2^	0.375	0.375	0.376	0.567	0.568

Control variables are as the same in Table 2. Standard errors are adjusted for firm-level clustering. *T*-statistics are presented in the parentheses. ***, **, * indicate statistical significance at the 1, 5, and 10% levels, respectively. BIGEVENT is an indicator for analyst visits that occur in the event window of major corporate events including mergers and acquisitions, seasoned equity offerings, right offerings, related party transactions, lawsuits, regulatory violations, and dividends. ADJACENT equals one for the visit events that are combined with visits with adjacent dates. ABSAS(−1) is the absolute abnormal returns on day − 1.

#### Considering treatment effect

One may worry that analysts self-select the way they visit listed companies, which will lead to endogeneity, i.e., treatment effect. So we use a two-step model to consider the possible treatment effect. In the first step, we regress *ONLINE* on companies’ location (*LOCATION*), size (*SIZE*), leverage (*LEVERAGE*), market to book value (*MB*), firm age (*LOGAGE*), profitability (*LOSS*), analyst coverage (*ANA*), and stock hold by funds (*FUND*). In the second step, we run Model 1 with Miller’s ratio (IMR) included. Results in Table 7 show that our main result still holds and the coefficient of IMR is significant at 10% level, indicating that the endogenous problem is not serious.

**Table 7:** Robustness check - Treatment effect.

(1) First step		(2) Second step	
	ONLINE		ABN_ABSAR(0,1)
LOCATION	0.219***	ONLINE	1.260***
	(4.98)		(2.73)
SIZE_t-1_	0.005	ABRET	−0.124***
	(0.17)		(−3.84)
ANA_t-1_	0.005***	ABRET_pre_visit	0.009
	(5.92)		(0.04)
Fund_t-1_	−0.000	TURNOVER	3.235***
	(−0.10)		(9.38)
MB_t-1_	−0.025**	MB_t-1_	−0.019*
	(−2.46)		(−1.72)
LEVERAGE_t-1_	−0.307**	LOGMV_t-1_	−0.058
	(−2.04)		(−1.57)
LOSS_t-1_	0.082	LOGCOVERAGE	0.037
	(0.97)		(1.45)
LOGAGE	−0.023	LEVERAGE_t-1_	−0.097
	(−0.56)		(−0.64)
		∆EPS_t-1_	−0.003
			(−0.27)
		SGROWTH_t-1_	−0.048
			(−0.73)
		BETA	−0.304***
			(−2.70)
		LOGAGE	−0.001
			(−0.02)
		IMR	−0.496*
			(−1.74)
_cons	−0.278		0.656
	(−0.50)		(1.26)
YEAR	YES	YEAR	YES
*N*	3,951	N	3,951
Pseudo-*R*^2^/	0.017	P > chi2	0.000

*T*-statistics are presented in the parentheses. ***, **, * indicate statistical significance at the 1, 5, and 10% levels, respectively. LOCATION is an binary variable which equals 1 when the firm is located in top 4 big cities and equals 0 otherwise. SIZE is the logarithm of 1 plus total assets. ANA is the number of analysts covering the firm. FUND is the percentage of stock hold by institutional investors. LOSS is an indicator that equals 1 when a firm’s net income is lower than 0.

## Further analyses

### Mechanism analyses

In this section, we test the possible mechanisms discussed in section 2 that make online visits more informative than offline visits during COVID-19.

#### Task orientation

In section 2, we propose that online visits are more likely to be task-oriented and participants are more likely to focus on the communication contents during online visits. Based on that, we construct two variables to capture the discussion depth. One is the length of questions asked (*QUESTION_length*) and the other is the length of answers (*ANSWER_length*). Results are shown in Columns (1) and (2) of Table 8. Both coefficients of ONLINE are statistically significant, indicating that analysts ask longer questions and managers answer in more words during online visits. We also conjecture that analysts ask more finance-related questions during online visits and use the number of finance-related questions (*FINANCEQ_num*) and the proportion of finance-related questions (*FINANCEQ_per*) as proxies. Columns (3) and (4) of Table 8 show that analysts ask more finance-related questions, both from number and percentage perspectives. All results indicate that participants focus more on the communication itself during online visits and online visits are more task-oriented.

**Table 8:** Mechanism Tests -Task orientation.

	(1)	(2)	(3)	(4)
	OLS regression		Poisson regression	
	QUESTION_length	ANSWER_length	FINANCEQ_num	FINANCEQ_per
ONLINE	0.098*	0.087**	0.087**	0.021*
	(1.88)	(2.49)	(2.50)	(1.76)
LOGMV	0.095	0.020	−0.007	−0.036***
	(1.10)	(0.51)	(−0.14)	(−2.82)
LEVERAGE	−0.155	−0.089	0.216	0.166***
	(−0.83)	(−0.54)	(1.44)	(2.94)
ROA	−0.165	0.024	0.215	0.097
	(−0.38)	(0.08)	(0.68)	(0.84)
∆EPS	−0.015	−0.001	−0.002	0.000
	(−1.07)	(−0.13)	(−0.22)	(0.11)
SGROWTH	0.148***	0.119***	0.034	0.004
	(2.87)	(2.82)	(0.72)	(0.25)
SALEVOL	0.245	0.192	−0.026	−0.017
	(1.46)	(1.51)	(−0.22)	(−0.46)
LOGAGE	−0.192*	−0.027	−0.000	0.043**
	(−1.83)	(−0.49)	(−0.00)	(2.45)
FUND	0.009*	0.005	0.006	−0.000
	(1.75)	(1.08)	(1.58)	(−0.20)
BIG4	−0.391***	−0.354***	−0.300**	−0.028
	(−3.18)	(−2.87)	(−2.43)	(−0.57)
LOGCOVERAGE	−0.073*	−0.073***	−0.043*	−0.002
	(−1.75)	(−3.61)	(−1.67)	(−0.33)
_cons	4.526***	7.428***	1.299*	0.968***
	(3.90)	(13.68)	(1.83)	(5.31)
INDUSTRY	YES	YES	YES	YES
YEAR	YES	YES	YES	YES
*N*	3,795	3,795	3,795	3,795
*R*^2^/Pseudo*R*^2^	0.090	0.091	0.013	0.076

Standard errors are adjusted for firm-level clustering. *T*-statistics are presented in the parentheses. ***, **, * indicate statistical significance at the 1, 5, and 10% levels, respectively. Control variables in Panel B are the same as in Table 2. Market value (LOGMV), firm leverage (LEVERAGE), return on asset (ROA), the change in net income (∆EPS), sales growth (SGROWTH), sales volatility (SALEVOL), firm age (LOGAGE), fund ownership (FUND), audit quality (BIG4), and analyst coverage (LOGCOVERAGE) are controlled.

#### Social class pressure

The second mechanism we proposed in Section 2 is that online visits reduce participants’ social class pressure and enable the disadvantaged ones to express more, resulting in higher communication efficiency. If this is true, we would observe the advantage of online visits to be greater when there are more visitors. So we divide the sample into more and fewer visitors sub-samples and run sub-sample regressions to test the impact of social class pressure. Results are presented in Table 9. All coefficients of *ONLINE* are significantly positive and the differences between coefficients in sub-samples are significant at 5%, suggesting that information sharing of online visits is better when more institutional investors participate, that is when social class pressure is higher.

**Table 9:** Mechanism Tests - Social class pressure.

	ABN_ABSAR(0,1)	ABN_ABSAR(0,1)	ABN_ABSAR(0,1)	ABN_ABSAR(0,1)
	num > 25	num < =25	num > 20	num < =20
	(1)	(2)	(3)	(4)
ONLINE	0.781***	0.243**	0.669***	0.248**
	(2.79)	(2.53)	(2.98)	(2.39)
***p*-value**	**0.0219**		**0.0350**	
ABRET	−0.185	−0.178	−0.167	−0.161
	(−1.08)	(−1.31)	(−1.11)	(−1.17)
ABRET_pre_visit	0.270	−0.510	0.201	−0.560
	(0.25)	(−1.27)	(0.22)	(−1.32)
TURNOVER	7.401*	7.430***	8.169**	7.756***
	(1.78)	(4.18)	(2.15)	(4.26)
MB_t-1_	−0.112	−0.034	−0.111	0.001
	(−0.62)	(−0.59)	(−0.62)	(0.03)
LOGMV_t-1_	−0.450	−0.027	−0.444	−0.094
	(−0.52)	(−0.08)	(−0.53)	(−0.27)
LOGCOVERAGE	−0.027	0.052	0.006	0.082
	(−0.08)	(0.59)	(0.02)	(0.89)
LEVERAGE_t-1_	−2.777	−0.410	−2.595	−0.543
	(−0.98)	(−0.34)	(−0.91)	(−0.45)
∆EPS_t-1_	0.003	−0.050*	−0.009	−0.054*
	(0.04)	(−1.88)	(−0.10)	(−1.88)
SGROWTH_t-1_	0.431	−0.029	0.411	0.013
	(0.77)	(−0.17)	(0.83)	(0.07)
BETA	1.233	0.681*	0.877	0.675
	(1.10)	(1.66)	(0.77)	(1.64)
LOGAGE	4.194	−0.116	5.023	−0.436
	(0.92)	(−0.09)	(1.23)	(−0.37)
_cons	−2.380	−0.479	−4.137	1.145
	(−0.16)	(−0.08)	(−0.28)	(0.19)
FIRM	YES	YES	YES	YES
YEAR	YES	YES	YES	YES
*N*	794	3,226	958	3,062
*R* ^2^	0.418	0.356	0.419	0.368

Standard errors are adjusted for firm-level clustering. *T*-statistics are presented in the parentheses. ***, **, * indicate statistical significance at the 1, 5, and 10% levels, respectively. Control variables in Panel B are the same as in Table 2.

#### Information dissemination

The third mechanism we put forward in Section 6 is that online visits amplify information dissemination. To test this, we compare institutional investors’ participation in online and offline visits in Table 10. We find that online visits are significantly and positively associated with institutions’ participation (*INSTITUTION_num*). Specifically, the number of funds (*FUND_num*), asset management firms (*AMC_num*), and insurance firms (*INSURANCE_num*) are all positively related to *ONLINE*. The effect is not only statistically significant but also economically significant. Taking the impact of *ONLINE* on the number of total institutions (*INSTITUTION_num*) as an example, the number of participating institutions in online visits is 26 more than that in offline visits. These results suggest that online visits attract more investors to participate, which amplifies information dissemination. Combined with the results in column (2) of Table 5, it can be seen that online visits make information dissemination more widely, which in turn affects the stock price.

**Table 10:** Mechanism Tests - Information dissemination.

	(1)	(2)	(3)	(4)
	Poisson regression			
	INSTITUTION_num	FUND_num	AMC_num	INSURANCE_num
ONLINE	1.118***	1.248***	1.197***	1.454***
	(16.12)	(17.26)	(15.25)	(13.56)
LOGMV	0.397***	0.262***	0.261***	0.396***
	(4.12)	(3.19)	(3.20)	(4.44)
LEVERAGE	0.040	−0.286	−0.278	−0.515
	(0.12)	(−0.83)	(−0.72)	(−1.18)
ROA	−0.373	−0.844	−0.844	−0.880
	(−0.60)	(−1.51)	(−1.17)	(−1.30)
∆EPS	−0.027	−0.008	−0.019	−0.012
	(−1.15)	(−0.27)	(−0.59)	(−0.54)
SGROWTH	0.035	−0.071	−0.105	−0.065
	(0.27)	(−0.59)	(−0.78)	(−0.54)
SALEVOL	0.181	0.150	0.205	0.074
	(0.92)	(0.81)	(0.95)	(0.32)
LOGAGE	−0.272***	−0.174*	−0.179	−0.166
	(−2.76)	(−1.74)	(−1.53)	(−1.41)
FUND	0.026***	0.031***	0.030***	0.030***
	(3.85)	(4.58)	(4.50)	(3.77)
BIG4	−0.392	−0.209	−0.306	−0.333
	(−1.19)	(−0.58)	(−0.74)	(−0.93)
LOGCOVERAGE	0.068	0.073	0.091	0.083
	(1.40)	(1.40)	(1.60)	(1.27)
_cons	−4.817***	−4.211***	−4.893***	−10.977***
	(−2.88)	(−3.11)	(−3.74)	(−6.88)
INDUSTRY	YES	YES	YES	YES
YEAR	YES	YES	YES	YES
*N*	3,795	3,795	3,795	3,795
Pseudo *R*^2^	0.370	0.287	0.269	0.215

Standard errors are adjusted for firm-level clustering. *T*-statistics are presented in the parentheses. ***, **, * indicate statistical significance at the 1, 5, and 10% levels, respectively. Control variables in Panel B are the same as in Table 2. Market value (LOGMV), firm leverage (LEVERAGE), return on asset (ROA), the change in net income (∆EPS), sales growth (SGROWTH), sales volatility (SALEVOL), firm age (LOGAGE), fund ownership (FUND), audit quality (BIG4), and analyst coverage (LOGCOVERAGE) are controlled.

### Prediction ability

To go a step further to test the information content of online visits, we examine the prediction effect of online visits on the absolute change in funds’ holding of the visited firms (∆*FUND*) and firms’ future performance (*∆EPS_q + 1_* and *SGROWTH_q + 1_*) following Jung et al. (2015). Results in Table 11 show that the number of online visits is positively related to funds’ absolute holding change and firms’ future performance, supporting that online visits provide additional information. During the COVID-19 period, online visit is not only the main reference for changes in fund holdings but also has a marginal predictive effect on the future operating performance of listed companies.

**Table 11:** Prediction effects.

	(1)		(1)	(2)
	∆FUND		∆EPS_q + 1_	SGROWTH_q + 1_
ONLINEnum	0.315*	ONLINEana	0.064**	0.016**
	(1.77)		(2.48)	(2.56)
OFFLINEnum	0.039	OFFLINEana	0.044***	0.009***
	(0.31)		(4.32)	(3.44)
LOGMV	0.050	LOGMV	−0.006	−0.004
	(0.43)		(−0.59)	(−1.55)
MB	0.154***	MB	0.007***	0.006***
	(2.73)		(2.66)	(8.67)
LEV	−0.734*	ROA	0.899***	−0.037
	(−1.75)		(3.44)	(−0.57)
∆EPS	0.038	LEVERAGE	0.102**	0.033***
	(1.59)		(2.30)	(2.73)
SGROWTH	0.257	PASTRET	0.108***	0.011*
	(1.29)		(3.82)	(1.69)
LOGAGE	−0.855***	∆EPS_q_	0.467***	
	(−4.57)		(33.61)	
LOGCOVERAGE	0.467***	SGROWTH_q_		0.690***
	(5.33)			(81.48)
RETURN_t_	1.181***			
	(5.50)			
RETURN_t-1_	0.294			
	(1.13)			
_cons	1.767	_cons	0.001	0.055
	(1.09)		(0.00)	(1.43)
YEAR	YES	YEAR	YES	YES
*N*	2,354	*N*	18,472	18,470
*R* ^2^	0.184	Adj. *R*^2^	0.252	0.583

Standard errors are adjusted for firm-level clustering. *T*-statistics are presented in the parentheses. ***, **, * indicate statistical significance at the 1, 5, and 10% levels, respectively.

## Conclusion

Technology development has changed the way we work and COVID-19 speeds it up. Remote work is a big challenge for specialists like financial analysts as their main job is to acquire information. We study the market reaction to analysts’ online and offline visits and find that online visits are more informative than offline visits after COVID-19. Compared with offline visits, online visits have obvious task-oriented advantages. In the case of bigger social class pressure, online visits have smore significant impacts on stock prices. Online visits enable more institutional investors to join in and make information spread more widely, which in turn has a greater impact on stock prices. Online visits can also predict changes in fund holdings and future company performance.

Our study implies that new technology helps decrease the damage of epidemics to the information environment of financial markets. However, due to the limitation of data availability, only Chinese data are used, which restricts the depth and extension of our paper. We believe that the impacts of culture, government policy, network infrastructure, and other factors on remote work efficiency are interesting and important research questions. In the future, these questions should be studied when data from other countries are available.

## Data availability statement

Publicly available datasets were analyzed in this study. This data can be found at: https://www.gtarsc.com.

## Author contributions

WJJ: conceptualization, software, data curation, writing—original draft, writing—review and editing, and funding acquisition. YYX: formal analysis, investigation, writing—original draft, and writing—review and editing. XWL: investigation, validation, and writing—review and editing. All authors contributed to the article and approved the submitted version.

## Funding

Shanghai Planning Project of Philosophy and Social Science [grant number 2019EJB004] and National Social Science Foundation [grant number 22CGL012].

## Conflict of interest

The authors declare that the research was conducted in the absence of any commercial or financial relationships that could be construed as a potential conflict of interest.

## Publisher’s note

All claims expressed in this article are solely those of the authors and do not necessarily represent those of their affiliated organizations, or those of the publisher, the editors and the reviewers. Any product that may be evaluated in this article, or claim that may be made by its manufacturer, is not guaranteed or endorsed by the publisher.

## Figures and Tables

**Table:** 

Variable	Definition
ABN_ABSAR(0,1)	The difference between the absolute 2-day three-factor-adjusted cumulated abnormal returns in the event period and the mean of the absolute rolling 2-day three-factor-adjusted cumulated abnormal returns in the estimation period, divided by the standard deviation of the mean absolute rolling 2-day three-factor-adjusted cumulated abnormal returns, where the portfolio market return is calculated with equal weight.
ONLINE	A dummy variable that equals one when the analyst visit is conducted online, and zero otherwise.
ABRET	The buy and hold market adjusted return from one year prior to the visit until 30 days before the visit.
ABRET_pre_visit	The absolute value of the buy and hold market adjusted return from one month prior to the visit until 11 days before the visit.
TURNOVER	The average monthly share turnover, measured as volume divided by shares outstanding, for the year prior to the visit until 30 days before the visit.
MB	The market-to-book ratio for the fiscal year prior to the visit.
LOGMV	The natural logarithm of the market value of the firm in the year prior to analyst visit (year t-1).
LOGCOVERAGE	The natural logarithm of one plus the number of analysts following the firm in the year of analyst visit.
LEVERAGE	The leverage ratio in the fiscal year prior to the visit, defined as the ratio of total debt divided by total assets.
∆EPS	The change in net income in year t-1, divided by net income in year t-2.
SGROWTH	The sales growth for the fiscal year prior to the visit, calculated as the ratio of total sales in the prior year divided by total sales in year t-2.
BETA	The stock beta (systematic risk), calculated over the period from day 420 to day 221 prior to the visit.
LOGAGE	The natural logarithm of one plus the number of years the company has been listed up to the fiscal year prior to the visit.
QUESTION_length	The natural logarithm of one plus the words of all questions asked.
ANSWER_length	The natural logarithm of one plus the words of all answers.
FINANCEQ_num	The number of financial-related questions.
FINANCEQ_per	The percentage of financial-related questions.
INSTITUTION_num	The number of institutions participating in the corporate visit.
FUND_num	The number of funds participating in the corporate visit.
AMC_num	The number of asset management companies participating in the corporate visit.
INSURANCE_num	The number of insurances participating in the corporate visit.
∆FUND	The absolute change of stocks held by funds of the visited firms per semi-year.
∆EPS_q+1_	The change in net income in the first quarter after the visit, divided by net income in the same quarter in prior year.
SGROWTH_q+1_	The ratio of total sales in the first quarter after the visit divided by total sales in the same quarter in prior year.
ONLINEnum	The natural logarithm of one plus the number of online visits to a firm in a semi-year.
OFFLINEnum	The natural logarithm of one plus the number of offline visits to a firm in a semi-year.
ONLINEana	The natural logarithm of one plus the number of analysts visited a firm online in a month.
OFFLINEana	The natural logarithm of one plus the number of analysts visited a firm offline in a month.
RETURN	The yearly stock return in year t.
PASTRET	The return over the three-month period [m-3, m-1], where m is the month in which the visits occur. Other variables are as defined before.

## Footnotes

^1^ https://www.gtarsc.com/

^2^As more investors participate in online visits, herding is more likely to happen around online visits.

## Appendix

Definition of Variables
